# Improvement of Geotechnical Properties of Clayey Soil Using Biopolymer and Ferrochromium Slag Additives

**DOI:** 10.3390/polym16101306

**Published:** 2024-05-07

**Authors:** Mustafa Yasin Çetin, Baki Bağrıaçık, Hatice Merve Annagür, Szymon Topoliński

**Affiliations:** 1 Department of Civil Engineering, Faculty of Engineering, Çukurova University, Adana 01330, Turkey; mstfacetin0@gmail.com; 2 Department of Civil Engineering, Faculty of Engineering, Toros University, Mersin 33140, Turkey; merve.cetin@toros.edu.tr; 3 Department of Road Engineering, Transport and Geotechnics, Faculty of Civil and Environmental Engineering and Architecture, Bydgoszcz University of Science and Technology, 85796 Bydgoszcz, Poland; szymon.topolinski@pbs.edu.pl

**Keywords:** biopolymer, ferrochromium slag, clay soil, geotechnical properties, soil improvement

## Abstract

The geotechnical properties of clay soil and its mixtures with different proportions (0.75%, 0.85%, 1%, and 1.15%) of Agar Gum biopolymer and Ferrochromium Slag (0.25%, 0.50%, 0.75%, and 1%), having various curing times and freeze-thaw cycles, were studied through a series of soil mechanical tests to investigate possibilities to improve its undesired/problematic plasticity, compaction, and shear strength characteristics. The results revealed that treatment with an optimal ratio of 1% Agar Gum and 1% Ferrochromium Slag alone, as well as together with, improved the geotechnical properties of the clay soil considerably. Both the unconfined and shear strength properties, along with the cohesion and internal friction angle, increased as much as 47 to 173%, depending on the curing time. The higher the curing time, the higher the shear strength, cohesion, and internal friction angle are up to 21 days. Deteriorating the soil structure and/or fabric, freeze-thaw cycles, however, seem to have an adverse effect on the strength. The higher the freeze-thaw cycle, the lower the shear strength, cohesion, and internal friction angle. Also, some improvements in the plasticity and compaction properties were determined, and environmental concerns regarding Ferrochromium Slag usage have been addressed.

## 1. Introduction

In recent years, continuous urban expansion movements and modernization efforts have left many construction sites with less ideal soil conditions for intended purposes, challenging geotechnical engineers in search of the most effective and economical soil improvement technique. Soil improvement is the alteration of any undesired property of a soil to improve its engineering performance, such as strength, stability, compressibility, compaction, plasticity, permeability, etc. Even in ancient times, people utilized crude but effective methods such as compaction, drainage, pre-loading, and consolidation and materials such as volcanic ash, mud, lime, bitumen, gypsum, and straw to improve the properties of soils [1,2,3,4].

After the Industrial Revolution and World War II, as cities overgrew, Portland cement, with low cost, high strength, and durability, emerged as a solution for soil stabilization [5,6,7,8,9]. Along with the early study of Terzaghi [9] on cement, subsequent studies on other admixtures such as lime, tire chips, fly ash, quarry dust, waste marble dust, stone dust, rice husk ash, sodium chloride, sodium hydroxide, calcium-based substances, and variable alkaline activated additives were also undertaken by various researchers for their potentials in soil improvement applications [10,11,12,13,14].

Though Ferrochromium Slag is produced worldwide by 12–16 million tons annually, as a byproduct of the metallurgical industry, it has recently been used in various civil engineering improvement applications such as in bricks, tiles/ceramics, mineral wool, asphalts/pavements, subgrades, cement, and concretes. It has not yet been used extensively for soil improvement purposes, even though it has some promising features [15,16,17,18,19]. The majority of Ferrochromium Slag is either discarded in huge piles across the landscape or landfilled in large volumes, which creates environmental concerns regarding the possibility of oxidation and the slow release of chromium to the environment. Therefore, utilization of these voluminous waste materials in an environmentally safe way remains a challenge for both the producers and potential users due to the leaching of chromium, especially the toxic and carcinogenic chromium (VI) form [20,21,22,23,24].

Due to environmental concerns (e.g., CO_2_ emission) related to cement and other conventional admixtures mentioned above involving chemical processes recently, biopolymers have increasingly been used for soil improvement purposes, and, consequently, a number of studies have been undertaken on various biopolymers for soil improvement [3,25,26,27,28]. Scientists have been trying to develop alternative methods that use more eco-friendly materials to enhance soil properties and reduce greenhouse gas emissions. Biopolymers derived from organics [29] emerged as a viable strategy as they modify soil structure, texture, or chemistry, enhancing cohesion, angle of internal friction, capillarity, permeability, elasticity, compressibility, and shear strength. However, they are not as effective as granular additives such as Ferrochromium Slag [30].

Though there are extensive studies on clay soil improvement using Agar Gum biopolymer (AG), there are limited studies on Ferrochromium Slag (FS) used for clayey soil improvement, and there are no studies utilizing both of them together; even though they have both separately demonstrated significant enhancements in geotechnical and environmental properties [31,32,33,34,35]. Also, there is no comprehensive study on mitigating the negative effects of chemical soil additives without sacrificing much of their effectiveness on soil improvement by using these ingredients in tandem with organic bio-friendly admixtures.

This study aimed to study the geotechnical properties of clay soil and its mixtures with different proportions of AG and FS alone, as well as together with, through a series of soil mechanical tests to investigate possibilities to improve its inadequate plasticity, compaction, and strength characteristics concerning various curing time and freeze-thaw cycles. The particular clayey soil used is named the Handere Formation of the late Miocene to Pliocene age [36], which outcrops throughout the northern parts of Adana city (the fifth largest city in Türkiye), where extensive settlement projects are now underway, especially after the devastating Pazarcık Mw = 7.8 (USGS) and Elbistan (Kahramanmaraş) Mw = 7.5 (USGS) so-called twin earthquakes on 6 February 2023 killing more than 60,000 people both in Türkiye and Syria. Concerns regarding the potential aforementioned environmental effects of FS usage have also been addressed. Using waste materials in geotechnical applications such as highway embankments, bridge abutments, and backfills behind retaining structures will positively impact the environment since massive quantities can be consumed in these voluminous structures.

## 2. Materials and Methods

### 2.1. Material

Though Handere formation predominantly comprises the studied clay soil levels, various other sedimentary rock types, such as sandstone, gravelly sandstone, siltstone, mudstone, and marl, are also included, especially in the upper levels of this geologically regressive unit.

Ferrochromium Slag (FS) is a waste product of the Ferrochromium production process, which involves smelting chromite ore with coke as a reducing agent in an electric arc furnace at a temperature of about 1500 °C. FS contains various amounts of chromium, iron, calcium, magnesium, aluminum, silicon, and other elements. FS has some mechanical properties that make it suitable for construction applications, such as aggregate, filler, or stabilizer. The Ferrochromium Slag (FS) used in this research was obtained from the manufacturer of ferrochromium metal in the Eti Krom A.Ş., Elazığ, Türkiye.

Agar Gum (AG) is a type of biopolymer that consists of complex sugar molecules called polysaccharides. AG has been used as a food additive for centuries as it can act as an emulsifier, thickener, stabilizer, flavor enhancer, and absorbent. AG can create gels that give texture and structure to food [37]. AG also has applications in other fields, such as microbiology, medicine, and dentistry [38]. AG has a neutral and inert backbone that does not react efficiently with other substances. Hence, it has been chosen to be used alongside FS for this study. The AG used in this research was obtained from KIMBIOTEK Chemical Company, İstanbul, Türkiye.

### 2.2. Sample Preparation and Testing

After being brought to the lab, the clay samples were identified and classified according to the Unified Soil Classification System (USCS) based on the ASTM D 2487 [39]. The liquid limit (LL), plastic limit (PL), and plasticity index (PI) of the samples were determined by following the ASTM D 4318 [40]. The specific gravities were determined following ASTM D 854-23 [41]. These parameters are important for index property analysis and for assessing soil behavior and consistency under different moisture conditions.

To evaluate the effectiveness of FS, the clay soil was mixed with different percentages (0.25%, 0.50%, 0.75%, 1%, and 1.15%) at the optimum water content obtained from the Proctor test performed according to [42]. Also, AG was added to the soil at three proportions (0.75%, 0.85%, 1%, and 1.15%) by weight and thoroughly mixed. The wet mixing method [43] was used to mix the biopolymers with the soil. The physical and mechanical properties of the soil–biopolymer mixtures were determined by conducting several laboratory tests, such as Atterberg limits, standard proctor compaction, unconfined compressive strength (UCS), and direct shear tests. The UCS and direct shear test specimens were cured for different durations (1, 7, 14, 21, and 28 days) to examine the effect of time on the strength gain.

The optimum water content for each instance of FS and AG content was determined using Proctor test results. The soil samples were prepared with optimum water content and different FS and AG biopolymer combinations. The samples were stored in sealed bags inside desiccators to prevent moisture loss. The unconfined compression tests were conducted after curing the samples for 1, 7, 14, 21, and 28 days, in accordance with the ASTM D 2166 [44]. The test results were compared with those of the untreated soil to assess the degree of improvement achieved by AG and FS combined.

The effect of FS and AG on the shear strength parameters of the clay soil, namely cohesion and internal friction angle, was investigated by mixing the clay soil with different proportions of the additives using shear box tests. The tests were run at a constant rate of shear displacement of 1 mm/min under varying vertical loads (3, 6, and 12 kg), simulating possible various overburden or field conditions. The tests were conducted according to the ASTM D 3080 [45].

Freeze-thaw damage is one of the main contributors to the deterioration of soil strength in cold climate areas. Deterioration caused by freeze-thaw cycling is primarily induced by pore water pressures within the pores of individual grains and between the grains, causing tensile cracking, which produces fine material and eventually weakens the soil strength. However, the studied clay soil outcrops in a mild-climate area below zero temperatures are reported during the 4–5-month periods in and around the winter seasons between 2010 and 2021 at the nearby meteorological stations (see [46] for example).

The samples prepared at determined optimum moisture contents were exposed to freeze-thaw cycling effects to study the degree of freeze-thaw deterioration on the soil strength. The samples were kept in desiccators to prevent changes in moisture content. The number of freeze-thaw cycles was 2, 4, 8, and 16, the temperatures were −20 °C for freezing and +25 °C for thawing, and the waiting time at each temperature was 6 h. The samples were wrapped with foil, placed in the freeze-thaw cabinet, and not removed from the cabinet during the test. After placing the samples in the freeze-thaw cabinet, they were brought to −20 °C and waited 6 h. Then, they were brought to +25 °C and waited another 6 h. This process, completed at the end of 12 h, was considered one cycle. The procedure followed here is similar to the ones used in some previous research [47,48,49,50,51]. After completing all cycles, unconfined compression and direct shear tests were performed on each sample.

## 3. Results and Discussions

The results of soil classification tests indicate that adding biopolymer slightly decreased the soil’s liquid limit (LL, from 42% to 40% at higher ratios of 1% and 1.15%) (Table 1). This means that the biopolymer increased the maximum water content, at which point the soil behaves as a liquid, but only marginally. The addition of biopolymer, however, did not seem to affect the PL and PI of the soil, which remained around 25% and 14–17%, respectively. This means that the biopolymer did not alter the minimum water content, at which point the soil becomes plastic (PL), or the range of water content over which the soil is plastic (PI).

According to the ASTM classification [39], the natural soil had moderate plasticity and shrinkage potential. The addition of biopolymer did not change these properties significantly as the soil remained moderately plastic. The biopolymer did not significantly impact the clay soil’s consistency and behavior, as measured by the Atterberg Limit test.

**Figure 1:**
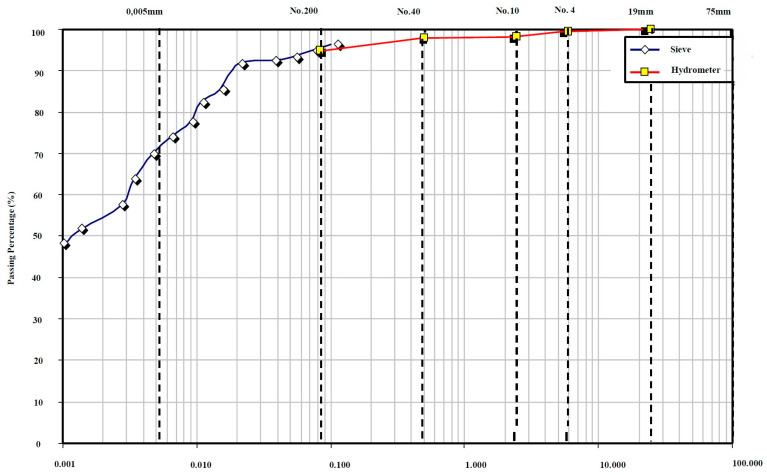
Grain size curves (sieve and hydrometer) for the clay soil.

**Figure 2:**
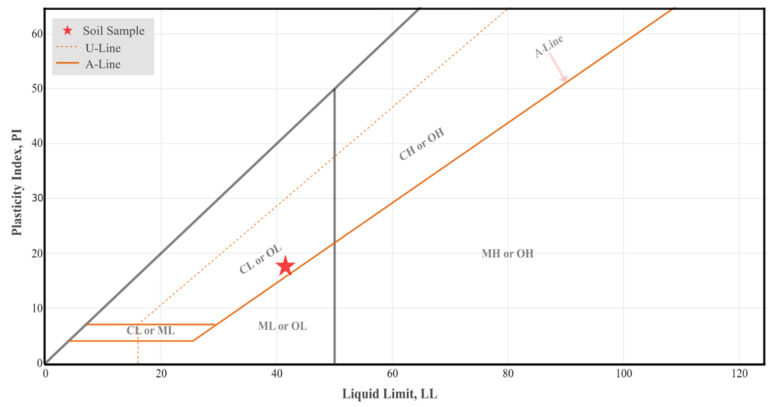
Classification of the pure clay soil on the Casagrande Plasticity Chart.

The washed sieve analysis and hydrometer test results are presented in the grain size distribution curve, as shown in Figure 1. The shape of the curve indicates a gradation of nearly “well-graded” grain size, and the soil sample consists of approximately 7% sand, 45.47% clay, and 47.30% silt. The grain size curve, along with the Casagrande plasticity chart (Figure 2), indicate that the clay soil class of the sample is “CL”, medium plastic inorganic clay according to the USCS. The clay types are probably mainly illites and some smectites as indicated on the modified plasticity chart of Casagrande [52] furnished by the data of Mitchell [53]. The pycnometer tests resulted in a specific gravity of 2.69.

The compaction test results are shown in Table 2, which summarizes the mean values of OMC and MDD for the natural soil and the soil mixed with different biopolymer ratios. The results indicated that adding AG biopolymer slightly reduced the OMC and increased the MDD of the soil. The OMC decreased from 17.75% for the natural soil to 17.25% for the soil mixed with 1% AG biopolymer, while the MDD increased from 1.834 g/cm^3^ for the natural soil to 1.861 g/cm^3^ for the soil mixed with 1% AG biopolymer. The results showed no significant difference in OMC and MDD among the different biopolymer ratios, except for the 1% AG biopolymer, which had a significantly higher MDD than the other ratios.

**Figure 3:**
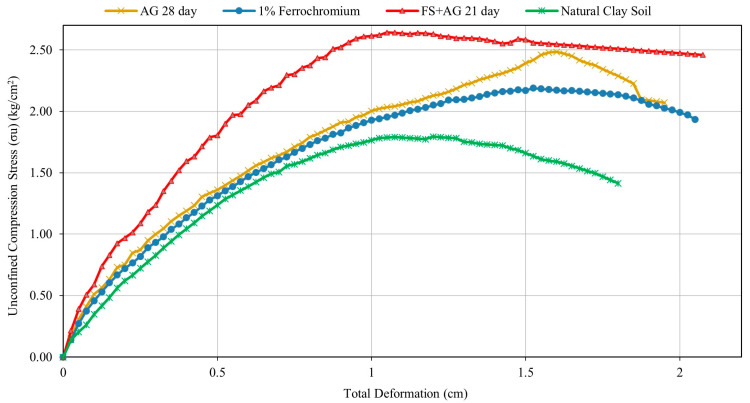
Typical UCS versus deformation curves for different admixtures under different treatments and the natural soil alone.

Figure 3 shows typical UCS versus deformation curves for the clay soil alone as well as different admixtures under different treatments. As seen in the figure, the unconfined compressive strength of the natural soil was 1.79 kg/cm^2^. Samples having a clear peak or failure point (brittle or mainly brittle/transition type deformation) on the UCS versus deformation curves seem to have failed between 11 and 15% axial deformation, demonstrating strain-softening behavior. Some samples, however, did not have a pronounced failure point, suggesting strain-hardening behavior. In such cases, the failure point was taken as the stress corresponding to 20% shear strain (upper limit), as recommended by ASTM D 2166-06 [44,54]. The addition of biopolymers and FS seems to have increased the strength of the soil. The higher the strength improvement, the higher the mixing ratios and extended curing periods. The biopolymers and FS seem to have formed strong bonds with the clay particles, increasing their strength.

**Figure 4:**
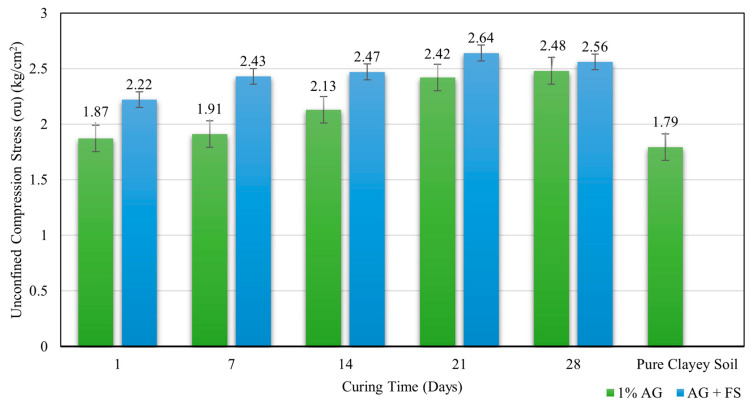
Compressive stress values of the soil treated with 1% AG and both 1% AG and 1% FS in relation to curing times.

The optimum AG content for soil improvement was found to be 1%, which increased the compressive strength values from 1.84 kg/cm^2^ at 1 day of curing time to 2.5 kg/cm^2^ at 28 days of curing time with the improvement percentages of 2.5% and 39%, respectively. This shows that the longer the curing time, the higher the compressive strength values due to the formation of stronger bonds between the AG biopolymer and soil particles. Figure 4 shows the variation of compressive strength with curing time at 1% AG content.

AG biopolymer’s maximum compressive strength values at 21 days of curing time were obtained at 0.85% and 1% AG content as 2.19 and 2.10 kg/cm^2^, respectively. These values were 22% and 17% higher than the natural soil compressive strength of 1.79 kg/cm^2^. The compressive strength values decreased at a higher AG content (1.15%), indicating an optimum AG content (1%) for soil improvement. The results are consistent with the previous studies which reported the positive effects of AG biopolymer on the engineering properties alternative to conventional chemical stabilizers for soil improvement in geotechnical engineering applications for clay soils.

**Figure 5:**
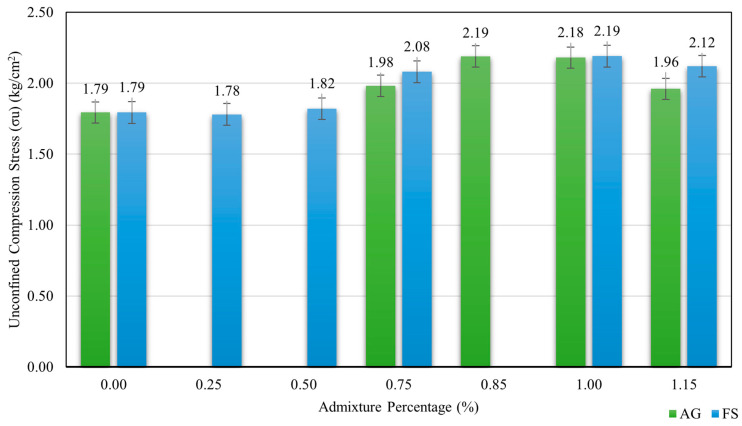
Compressive stress values of soil treated with various percentages of AG and FS.

The soil–FS mixtures showed an increase in compressive strength with an increasing FS content of up to 1%, reaching a peak value of 2.19 kg/cm^2^, which was 21.99% higher than the natural soil. This improvement may be explained by FS’s filling effect and pozzolanic reaction, which reduces the void ratio and increases the density of the soil. The soil–FS mixtures showed a decrease in compressive strength with a further increasing FS content beyond 1%, indicating an optimum FS content range for soil stabilization. The compressive strength values at 1.25%, 1.5%, 1.75%, and 2% FS content were lower than the natural soil, suggesting a negative effect of excessive FS on the soil structure. Figure 5 shows the variation of compressive strength with the FS content at various mixture ratios.

**Figure 6:**
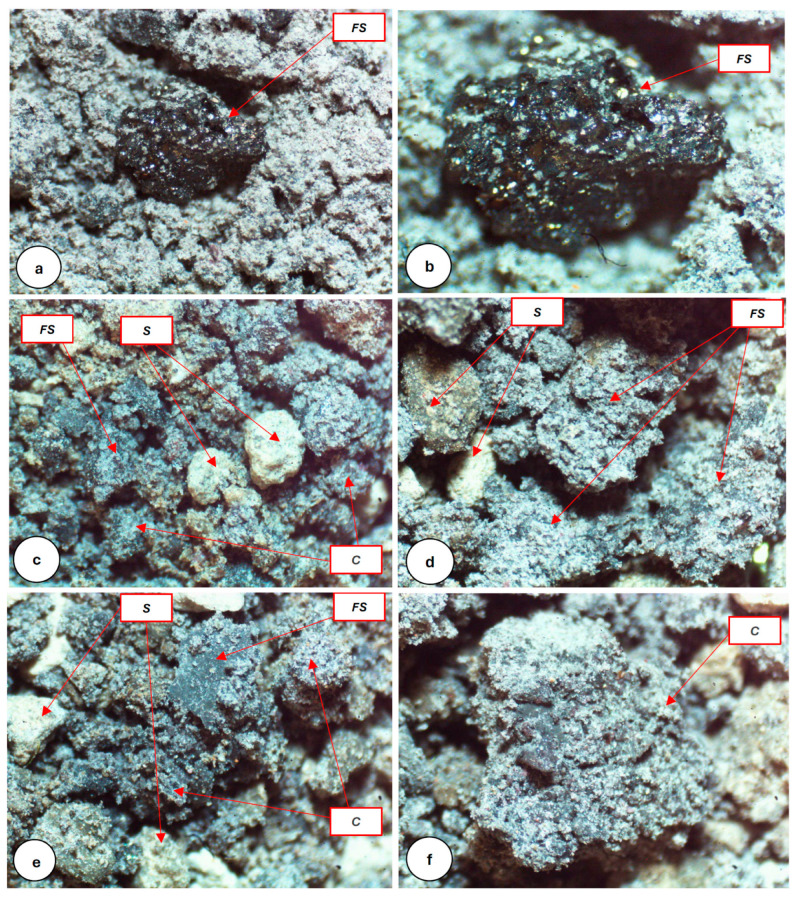
The microscopic views of (**a**) FS grain surrounded by silt grains and clay domains not treated with AG, (**b**) a close-up view of the exact FS grain, (**c**) a general view of the sample with both FS and AG admixtures together, (**d**) a close-up view of FS and AG admixtures together, (**e**) FS grain partially coated with AG, and (**f**) a clay domain coated with AG. Notice the uncoated (**a**,**b**), coated (**d**), and partially coated (**e**) nature of the FS grains surrounded by other constituents. FS: Ferrochromium Slag, S: silt, C: clay domain. Photo lengths: (**a**) 1.5 mm, (**b**,**f**) 1 mm, (**c**,**e**) 5 mm, and (**d**) 3 mm.

As previously explained, FS poses some environmental and health hazards, and consequently, FS needs to be treated before it can be used safely. Therefore, the feasibility of using AG biopolymer with FS for stabilizing clay soil was investigated. The hypothesis was that combining these additives would yield more improved mechanical properties of the soil, eliminating any potential effect of the FS without trading in much of its essential soil improvement abilities. Since the FS is in a granular form and the treated biopolymer in a gelatinous form, with adequate mixing, the gelatin matrix of the biopolymer would encase the FS grains, thus removing the direct contact with the soil and possible groundwater interaction, and therefore, eliminating or reducing potential environmental hazards related to chromium leaching. Previous studies on soil improvement using biopolymers utilized similar approaches for different admixtures. Figure 6 shows microscopic views of the soil structures of only FS-treated soil and FS + AG-treated soil. In only FS-treated soil, FS grains are easily seen in an uncoated nature, displaying a crystal-like form with a metallic luster (Figure 6a,b). In the FS + AG-treated soil, however, the FS grains, as well as the other constituents, are coated with AG biopolymer enclosing the FS grains, thus removing the direct contact with the soil and eliminating or reducing potential environmental hazards related to chromium leaching (Figure 6c–f).

Adding FS and AG together significantly improved the compressive strength of clay soil. The highest compressive strength value was achieved for 1% FS and 1% AG biopolymers after 21 days of curing time, as 2.64 kg/cm^2,^ which is 46.9% higher than the natural soil compressive strength of 1.79 kg/cm^2^. Figure 4 shows the variation of compressive strength with a combination of FS and AG biopolymers for different curing times. The enhancement in compressive strength was attributed to the synergistic effect of FS and biopolymers on the soil structure. FS seems to have functioned as a filler material and a pozzolanic agent, which reduced the void ratio and increased the density of the soil. AG biopolymer acted as a binder material and a water retention agent, improving the cohesion and stiffness of the soil.

Regarding the freeze-thaw performance, results show that the FS-stabilized samples showed the highest freeze-thaw resistance among the three types of stabilized samples, with compressive strength values ranging from 1.64 kg/cm^2^ to 1.39 kg/cm^2^ as the number of cycles increased from 2 to 16, indicating that FS is more effective than AG biopolymer in enhancing the freeze-thaw resistance of the clay soil. It seems to have acted as a filler material and a pozzolanic agent, reducing the void ratio and increasing the density of the soil.

The AG biopolymer-stabilized samples showed the lowest freeze-thaw resistance among the three types of stabilized samples, with compressive strength values ranging from 1.52 kg/cm^2^ to 0.98 kg/cm^2^ as the number of cycles increased from 2 to 16. This suggests that AG biopolymer is less effective than FS in enhancing the freeze-thaw resistance of clay soil, as it acted as a binder material and a water retention agent, which increased the cohesion and stiffness of the soil but also made it more susceptible to water absorption and ice expansion.

The optimum mixture-stabilized samples showed a moderate freeze-thaw resistance between the FS stabilized and AG biopolymer-stabilized samples, with compressive strength values ranging from 1.58 kg/cm^2^ to 1.08 kg/cm^2^ as the number of cycles increased from 2 to 16, the optimum mixture has a synergistic effect on enhancing the freeze-thaw resistance of clay soil, as it combined the benefits of both FS and AG biopolymer and reduced their drawbacks.

**Figure 7:**
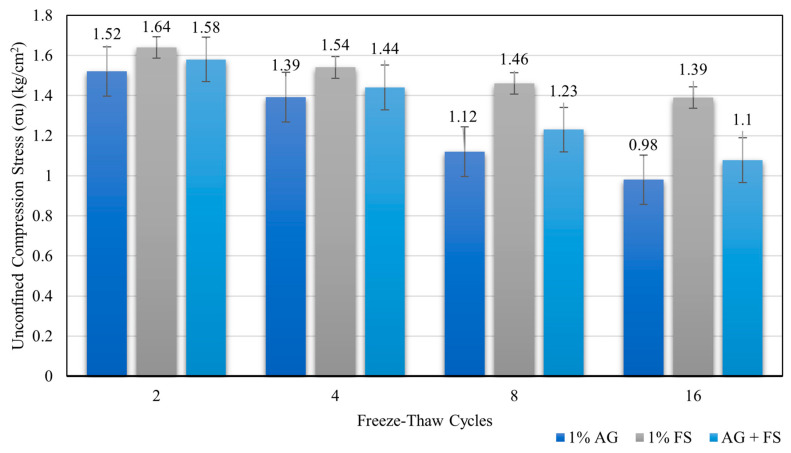
Compressive stress values of soil mixed with additives and subjected to various cycles of freeze-thaw procedure.

The compressive strength values of all the stabilized samples decreased with increasing cycles, indicating a gradual deterioration of the soil structure due to repeated freezing and thawing. Figure 7 shows the variation of compressive strength with the number of cycles for several types of stabilized samples.

**Figure 8:**
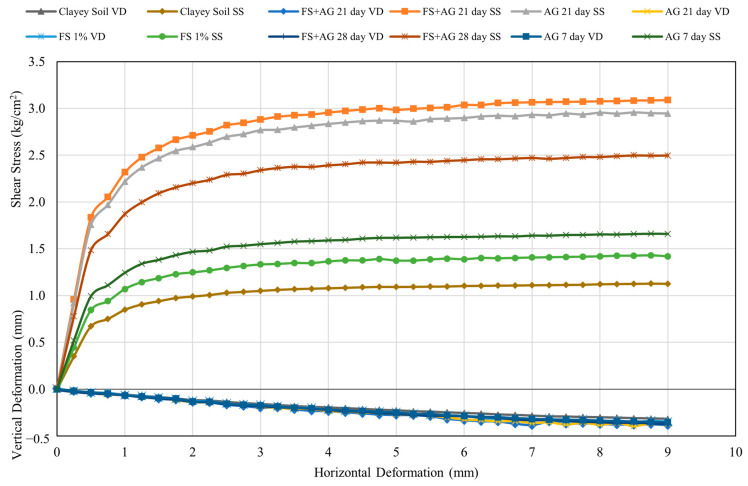
Typical shear stress versus deformation curves for the clay soil alone as well as with different admixtures under different treatments. SS: Shear stress, VD: Vertical deformation, FS: Ferrochromium Slag, AG: Agar Gum.

Figure 8 shows typical shear strength and vertical or volumetric strain characteristics of the clay soil alone as well as with different admixtures under different treatments. As seen in the figure, the shear strength of the natural soil was 1.13 kg/cm^2^. The shear stress parameters of cohesion and angle of internal friction were found to be 0.4 kg/cm^2^ and 11.5 degrees, respectively. Most of the samples did not have a clear or pronounced failure point exhibiting strain-hardening behavior, which is typical of cohesive soils like clays. Here, the failure point was taken as the stress corresponding to 15% shear strain as recommended by [54,55], and ASTM D 3080-04 [45]. Figure 8 also shows the vertical (volumetric) deformation changes as the samples were sheared under the selected normal pressures. As seen in the figure, the vertical deformation for the clay soil alone, as well as with different admixtures under different treatments, are all negative, or compression is taking place which is consistent with typical normally consolidated and remolded clayey soils.

**Figure 9:**
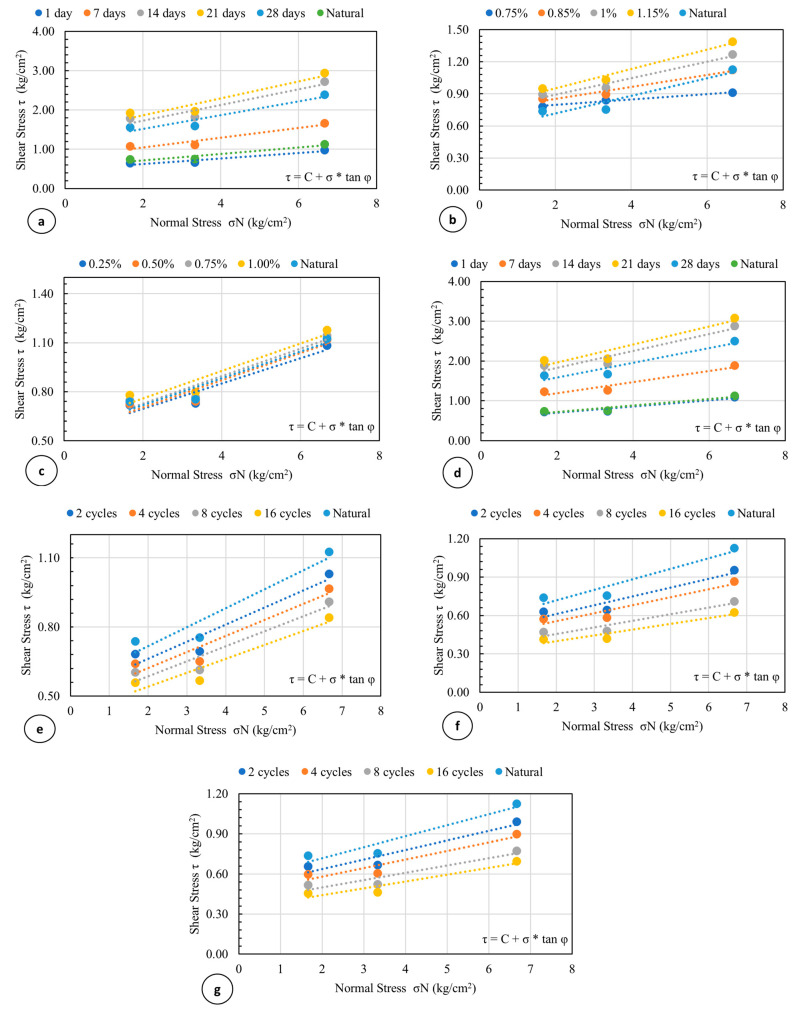
Failure envelopes of the soil mixed with (**a**) optimum 1% AG cured for different periods, (**b**) various percentages of AG cured for 21 days, (**c**) FS at various ratios, (**d**) both optimum 1% AG and 1% FS, (**e**) optimum 1% AG at various freeze-thaw cycles, (**f**) optimum 1% FS at various freeze-thaw cycles, and (**g**) both optimum 1% AG and FS at various freeze-thaw cycles. The failure envelope for the pure clay soil was also included for comparison. For easy differentiation, some of the envelope scales have been altered. The “ * ” symbol indicates multiplication.

**Figure 10:**
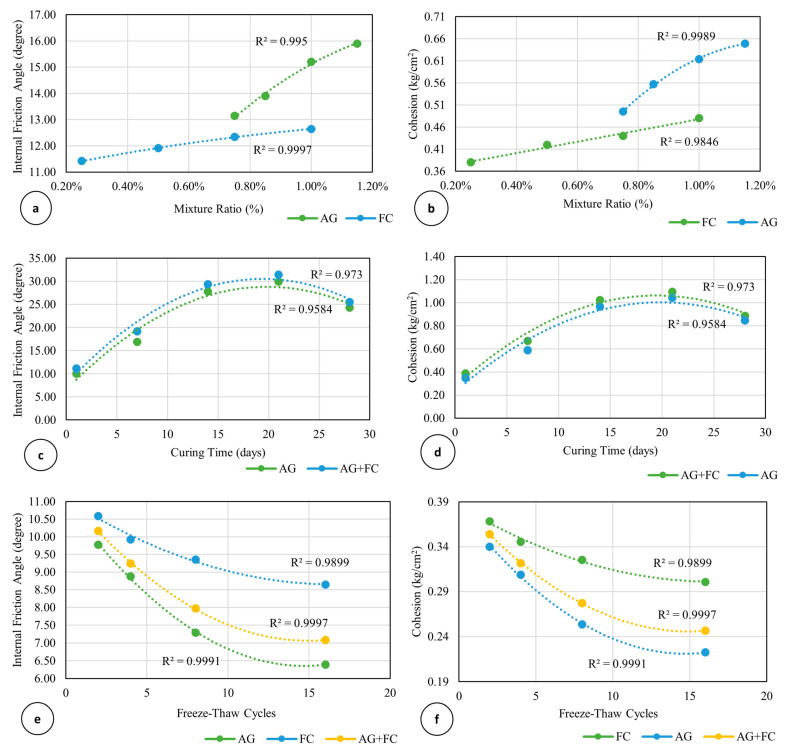
Comparison between (**a**) internal friction angle values by various FS and AG mixing ratios, (**b**) cohesion values by various FS and AG mixing ratios, (**c**) internal friction angle values by various FS and AG + FS curing times, (**d**) cohesion values by various FS and AG + FS curing times, (**e**) internal friction angles by freeze-thaw cycles for FS, AG and AG + FS, and (**f**) cohesion by freeze-thaw cycles for FS, AG and AG + FS.

The results showed that the addition of AG increased the shear strength parameters of cohesion and internal friction angles (Figure 9 and Figure 10). While the cohesion of the soil–biopolymer mixture ranged from 0.5 to 0.7 kg/cm^2^, the internal friction angle ranged from 13.5 to 15.5 degrees, which were initially 0.4 kg/cm^2^ and an internal friction angle of 11.5 degrees, respectively (Figure 9 and Figure 10). The optimal curing time for achieving maximum shear strength was 21 days, as it allowed sufficient time for the formation and hardening of hydrogels that bond the soil particles together. The interaction of AG and water forms the hydrogels, and they act as a binder material and a water retention agent for the soil. The shear strength of the soil–biopolymer mixture after 21 days of curing was determined as 2.64 kg/cm^2^, 46.9% higher than that of the native clay soil with 1.79 kg/cm^2^ shear strength.

The results indicate that the addition of FS increased the cohesion coefficient and the internal friction angle considerably (Figure 9 and Figure 10). The cohesion of the soil–slag mixture was determined as 0.5 kg/cm^2^, which is 25% higher than that of the natural clay soil. The internal friction angle of the soil–slag mixture was determined as 14.48 degrees, 25.9% higher than that of the natural clay soil. The addition of AG and FS also increased the clay soil’s cohesion and internal friction angle. The cohesion of the soil–slag–biopolymer mixture ranged from 0.39 kg/cm^2^ to 1.09 kg/cm^2^. At the same time, the internal friction angle varied from 11.17 degrees to 31.45 degrees, both of which are considerably higher than the pure clay soil values (Figure 9 and Figure 10).

Here again, the optimal curing time for achieving maximum shear strength was 21 days, as it allowed sufficient time for the formation and hardening of hydrogels that bond the soil particles together. The shear strength of the soil–slag–biopolymer mixture after 21 days of curing time was determined as 2.64 kg/cm^2^, which is 46.9% higher than that of the natural clay soil, having 1.79 kg/cm^2^ shear strength. The results also show that the shear strength behavior becomes more ductile under higher normal loads than that of the lower normal stress tests. The shear stresses under normal loads of 3 kg, 6 kg, and 12 kg ranged from 0.72 kg/cm^2^ to 2.02 kg/cm^2^, 0.73 kg/cm^2^ to 2.06 kg/cm^2^, and 1.09 kg/cm^2^ to 3.08 kg/cm^2^, respectively, all of which are higher than those of the natural clay soil ranging from 0.74 kg/cm^2^ to 1.13 kg/cm^2^, 0.76 kg/cm^2^ to 1.13 kg/cm^2^, and 1.01 kg/cm^2^ to 1.13 kg/cm^2^, respectively.

The freeze-thaw cycles decreased cohesion and internal friction angle (Figure 9 and Figure 10). While the cohesion of the soil–slag–biopolymer mixture varied from 0.22 kg/cm^2^ to 0.33 kg/cm^2^, the internal friction angle ranged from 6.39 to 9.35 degrees, both lower than those of the natural clay soil values.

## 4. Conclusions

The results revealed that both AG and FS alone, as well as together, considerably improved the geotechnical engineering properties of the clay soil. The following main conclusions were obtained.

The unconfined compressive strength of AG-treated soil increased up to 22%, indicating an optimum mixing ratio of 1% AG. An additional 17% increase is obtained after 28 days of curing time. The higher the curing time, the higher the unconfined compressive strength. Similarly, 1% FS treatment increased the unconfined compressive strength up to 22%. With the combination of FS and AG, as much as a 47% improvement after 21 days of curing is obtained. As for the freeze-thaw resistance, depending on the cycles, the unconfined compressive strengths of FS and AG-treated soils decreased as much as 22% and 45%, respectively. The higher the freeze-thaw cycle, the lower the unconfined compressive strength. The decrease for the combination of 1% FS and AG is about 40%, indicating that FS is more effective than AG in enhancing the freeze-thaw resistance of clay soil.

The shear strength, as well as the cohesion and the internal friction angle, increased as much as 160% for the 1% AG-treated soil depending on the normal pressure and curing time. The increase for the same parameters for the 1% FS-treated soil varied between 25 and 26%. However, for the combination of 1% FS and 1% AG, it was as much as 173% under the same conditions. The higher the normal pressure and curing time, up to 21 days, the higher the shear strength, cohesion, and internal friction angle. As for the freeze-thaw resistance, depending on the cycles, the shear strengths of the FS and AG-treated soils decreased as much as 26% and 45%, respectively. The decrease for the combination of the 1% FS and AG is about 39%, indicating that FS is more effective than AG in enhancing the freeze-thaw resistance of clay soil. The related shear strength parameters of cohesion and the internal friction angle decreased by similar percentages. The higher the freeze-thaw cycle, the lower the shear strength, cohesion, and internal friction angle.

Finally, the results indicate that the optimal mixtures of 1% AG biopolymer and 1% FS alone, as well as together, can be used above ground water tables where low permeability and high strength are needed in fills such as highway embankments, bridge abutments, and backfills behind retaining structures, especially when low bearing capacity and high settlement problems exist. Furthermore, the result of this study demonstrates that biopolymer and ferrochrome additives are economical and environmentally friendly methods, especially when used together. However, freeze-thaw cycles in cold seasons seem to have an adverse effect on the shear strength. The soil constituents and, in turn, the structure or fabric seem to deteriorate due to tensile cracking of the grains caused by pore water pressures within the pores of individual grains and between the grains themselves after each cycle. Though the aforementioned environmental concerns regarding Ferrochromium Slag usage have been addressed under microscopic conditions (i.e., relationships between the AG-treated FS grains and other constituents) in this study, the formation of hexavalent (VI) chromium requires additional research. Therefore, further studies are recommended.

## Figures and Tables

**Table 1:** LL, PL, and PI values for different additive mixture ratios.

AG (%)	Liquid Limit (LL)	Plastic Limit (PL)	Plasticity Index (PI)	USCS Classification
0% (Natural)	42.19%	25.63%	16.56%	CL
0.75%	40.63%	25.88%	14.75%	CL
0.85%	40.38%	25.97%	14.41%	CL
1.00%	40.13%	26.06%	14.07%	CL
1.15%	39.88%	26.15%	13.73%	CL
FS (%)				
0.25%	41.94%	25.69%	16.25%	CL
0.50%	41.69%	25.75%	15.94%	CL
0.75%	41.44%	25.81%	15.63%	CL
1.00%	41.19%	25.88%	15.31%	CL

**Table 2:** Standard Compaction test results.

AG (%)	Optimum Water Content (%)	Dry Unit Weight (g/cm^3^)
0 (clay soil)	17.50	1.83
0.75	17.12	1.84
0.85	17.04	1.86
1	16.85	1.87
1.15	16.74	1.88
FS (%)		
0.25	17.28	1.83
0.5	17.06	1.84
0.75	16.84	1.84
1	16.62	1.84

## Data Availability

Data are contained within the article.
